# Timing matters when correcting fake news

**DOI:** 10.1073/pnas.2020043118

**Published:** 2021-01-25

**Authors:** Nadia M. Brashier, Gordon Pennycook, Adam J. Berinsky, David G. Rand

**Affiliations:** ^a^Department of Psychology, Harvard University, Cambridge, MA 02138;; ^b^Paul J. Hill School of Business, University of Regina, Regina, SK S4S 0A2, Canada;; ^c^Kenneth Levene Graduate School of Business, University of Regina, Regina, SK S4S 0A2, Canada;; ^d^Department of Psychology, University of Regina, Regina, SK S4S 0A2, Canada;; ^e^Department of Political Science, Massachusetts Institute of Technology, Cambridge, MA 02139;; ^f^Sloan School, Massachusetts Institute of Technology, Cambridge, MA 02139;; ^g^Department of Brain and Cognitive Sciences, Massachusetts Institute of Technology, Cambridge, MA 02139

**Keywords:** fake news, misinformation, correction, fact-checking, memory

## Abstract

Countering misinformation can reduce belief in the moment, but corrective messages quickly fade from memory. We tested whether the longer-term impact of fact-checks depends on when people receive them. In two experiments (total *N =* 2,683), participants read true and false headlines taken from social media. In the treatment conditions, “true” and “false” tags appeared before, during, or after participants read each headline. Participants in a control condition received no information about veracity. One week later, participants in all conditions rated the same headlines’ accuracy. Providing fact-checks after headlines (*debunking*) improved subsequent truth discernment more than providing the same information during (*labeling*) or before (*prebunking*) exposure. This finding informs the cognitive science of belief revision and has practical implications for social media platform designers.

Concern about fake news escalated during the run-up to the 2016 US presidential election, when an estimated 44% of Americans visited untrustworthy websites (1). Faced with mounting public pressure, social media companies enlisted professional fact-checkers to flag misleading content. However, misconceptions often persist after people receive corrective messages (*continued influence effect*; ref. 2). Detailed corrections increase the likelihood of knowledge revision (3), but social media platforms prioritize user experience and typically attach simple tags (e.g., “disputed”) to posts. Can we optimize the longer-term impact of these brief fact-checks by presenting them at the right time?

There are arguments for placing fact-checks before, during, or after disputed information. Presenting fact-checks before headlines might confer psychological resistance. Inoculating people to weakened arguments makes them less vulnerable to persuasion (4). As examples, reading about “fake experts” protects people from climate science myths (5), and playing a game involving common disinformation tactics (e.g., faking an official Twitter account) helps people detect fake news (6). Prebunking could direct attention to a headline’s questionable features (e.g., sensational details). On the other hand, people might ignore the content entirely and miss an opportunity to encode it as “false.”

Alternatively, reading fact-checks alongside news could facilitate knowledge revision. Encoding retractions requires building a coherent mental model (7), which is easiest when misinformation and its correction are coactive (8). This mechanism explains why corrections rarely reinforce the original false belief (i.e., do not “backfire”) (9)—it is actually best to restate a myth when retracting it (10, 11). Thus, labeling a headline as “true” or “false” could increase salience and updating.

Finally, providing fact-checks after people process news could act as feedback, boosting long-term retention of the tags. Corrective feedback facilitates learning (12), especially when errors are made with high confidence (13). Prediction error enhances learning of new facts that violate expectations (14). Surprise also occurs when low-confidence guesses turn out to be right, improving subsequent memory (15). Debunking after readers form initial judgments about headlines could boost learning, even if they did not make an error.

Despite the extensive previous work on corrections, no study has directly compared the efficacy of equivalent corrections delivered before, during, or after exposure. In two nearly identical experiments (total *N =* 2,683), we tested whether the timing of corrections to fake news impacts discernment 1 wk later. Participants were exposed to 18 true and 18 false news headlines taken from social media (Fig. 1); they saw “true” and “false” tags immediately before (*prebunking*), during (*labeling*), or immediately after (*debunking*) reading and rating the accuracy of each headline. In a control condition, participants received no veracity information. One week later, they rated the accuracy of the 36 headlines again. To maximize power, we analyzed the final accuracy ratings from the two experiments together using linear regression with robust SEs clustered on subject and headline. We included dummies for each treatment condition, headline veracity (0 = false, 1 = true), and study. We also included the interaction between veracity and the treatment dummies, and the interaction between veracity and the study dummy.

**Fig. 1.**
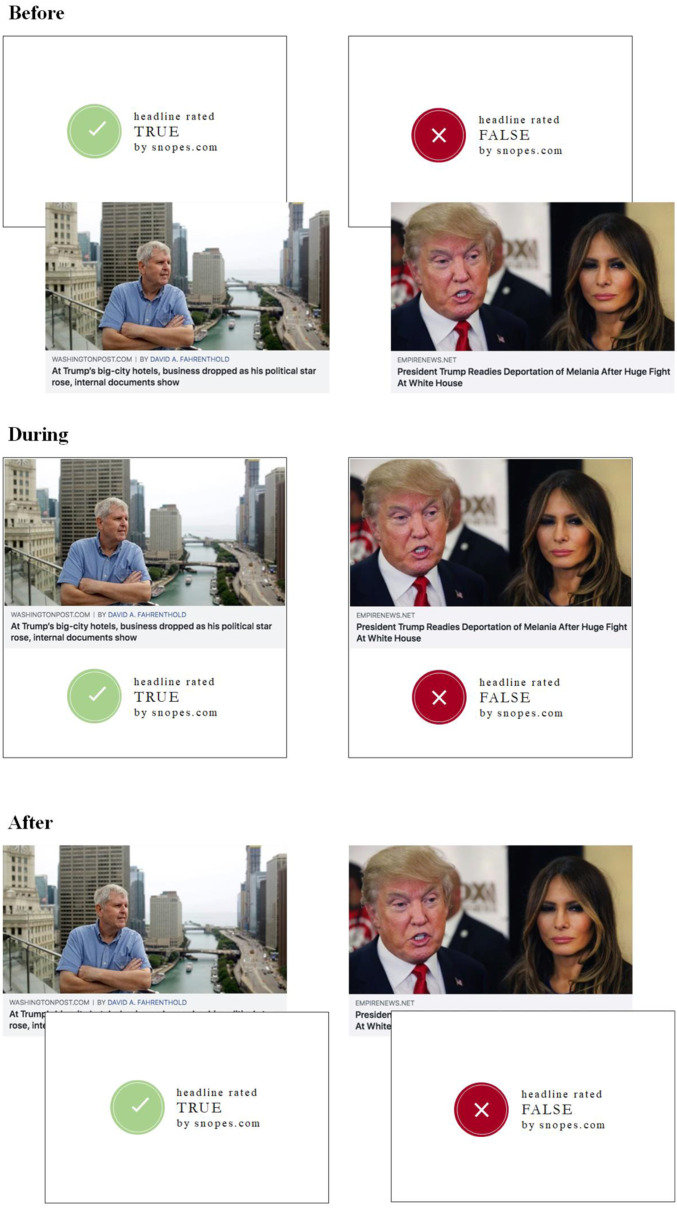
Sample true and false headlines, as shown in the before, during, and after conditions. Fact-checks appeared on separate screens in the before and after conditions.

## Results

**Fig. 2.**
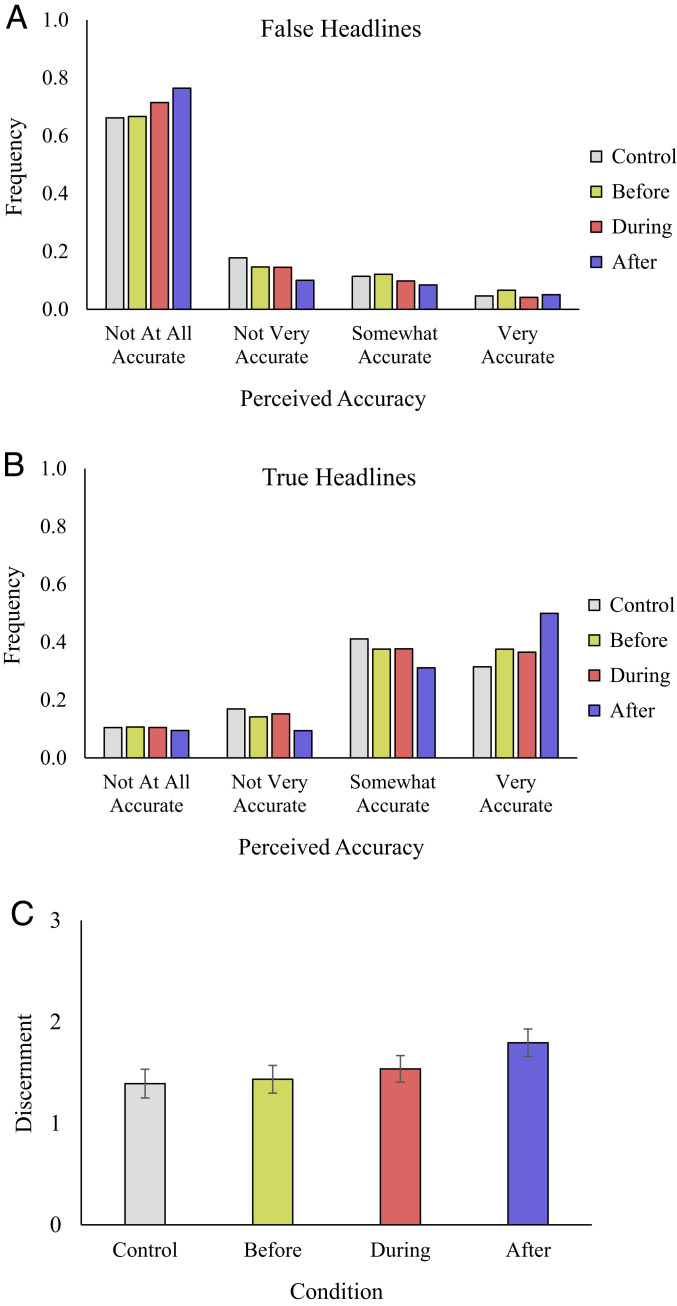
Distribution of accuracy ratings for false (*A*) and true (*B*) headlines and discernment (*C*) 1 wk after exposure, by treatment. Error bars indicate 95% CIs.

Fig. 2*A* shows the distribution of accuracy ratings for false headlines after 1 wk. Presenting corrections after, *b* = −0.123, *F*(1, 96587) = 16.34, *P* < 0.001, *P*_stan_ < 0.001, and during, *b* = −0.081, *F*(1, 96587) = 7.45, *P* = 0.006, *P*_stan_ = 0.033, exposure to each headline decreased belief in false headlines relative to the control condition (to a similar extent, *F*(1, 96587) = 2.03, *P* = 0.154). Presenting corrections before exposure, conversely, did not significantly reduce belief in false headlines, *b* = 0.042, *F*(1, 96587) = 1.74, *P* = 0.188, and was less effective than presenting corrections after, *F*(1, 96587) = 25.39, *P* < 0.001, *P*_stan_ < 0.001, or during, *F*(1, 96587) = 15.11, *P* < 0.001, *P*_stan_ < 0.001, reading.

Fig. 2*B* shows the distribution of accuracy ratings for true headlines after 1 wk. While all three treatments significantly increased belief in true headlines relative to the control condition (*F*(1, 96587) > 6.75, *P* < 0.01, *P*_stan_ < 0.05 for all), presenting corrections after exposure was significantly more effective than during, *F*(1, 96587) = 65.53, *P* < 0.001, *P*_stan_ < 0.001, or before, *F*(1, 96587) = 47.02, *P* < 0.001, *P*_stan_ < 0.001, exposure.

Fig. 2*C* shows that this leads to significantly greater truth discernment (the difference in belief between true and false headlines) when corrections appeared after compared to during, *F*(1, 96587) = 37.74, *P* < 0.001, *P*_stan_ < 0.001, or before, *F*(1, 96587) = 65.08, *P* < 0.001, *P*_stan_ < 0.001, exposure (and during was marginally more effective than before, *F*(1, 96587) = 6.33, *P* = 0.012, *P*_stan_ = 0.062). Although before was more effective in Experiment 2 than in Experiment 1, after is still more effective than during or before exposure when considering each experiment separately (*P* < 0.001, *P*_stan_ < 0.01 for all comparisons).

Interestingly, neither analytic thinking, as measured by the Cognitive Reflection Test, nor political knowledge moderated the treatment effects (*P*s > 0.421), despite both measures being associated with better baseline discernment (*P* < 0.001, *P*_stan_ < 0.001 for both). Lastly, providing corrections after reading may have been less effective for headlines that aligned with participants’ partisanship than for headlines that did not, *F*(1, 96587) = 5.06, *P* = 0.025, *P*_stan_ = 0.129, while the effectiveness of during and before did not differ based on partisan alignment (*P*s > 0.30). Nonetheless, after was more effective than before or during exposure even for politically aligned headlines (*P* < 0.001, *P*_stan_ < 0.001, for all comparisons).

For regression tables and separate analyses of each experiment, see Open Science Framework (OSF, https://osf.io/bcq6d/).

## Discussion

We found consistent evidence that the timing of fact-checks matters: “True” and “false” tags that appeared immediately after headlines (debunking) reduced misclassification of headlines 1 wk later by 25.3%, compared to an 8.6% reduction when tags appeared during exposure (labeling), and a 6.6% increase (Experiment 1) or 5.7% reduction (Experiment 2) when tags appeared beforehand (prebunking).

These results provide insight into the continued influence effect. If misinformation persists because people refuse to “update” beliefs initially (16), prebunking should outperform debunking; readers know from the outset that news is false, so no updating is needed. We found the opposite pattern, which instead supports the *concurrent storage hypothesis* that people retain both misinformation and its correction (17); but over time, the correction fades from memory (e.g., ref. 18). Thus, the key challenge is making corrections memorable. Debunking was more effective than labeling, emphasizing the power of feedback in boosting memory.

Our implementation models real-time correction by social media platforms. However, delivering debunks farther in time from exposure may be beneficial, as delayed feedback can be more effective than immediate feedback (19). Similarly, while our stimulus set was balanced, true headlines far outnumber false headlines on social media. Debunking may improve discernment even more when “false” tags are infrequent, as they would be more surprising and thus more memorable (15). On the other hand, mindlessly scrolling, rather than actively assessing accuracy at exposure, may lead to weaker initial impressions to provide feedback on, thereby reducing the advantage of debunking over labeling.

Ideally, people would not see misinformation in the first place, since even a single exposure to a fake headline makes it seem truer (20). Moreover, professional fact-checkers only flag a small fraction of false content, but tagging some stories as “false” might lead readers to assume that unlabeled stories are accurate (*implied truth effect*; ref. 21). These practical limitations notwithstanding, our results emphasize the surprising value of debunking fake news after exposure, with important implications for the fight against misinformation.

## Materials and Methods

We selected 18 true headlines from mainstream news outlets and 18 false headlines that Snopes.com, a third-party fact-checking website, identified as fabricated (Fig. 1). The Committee on the Use of Human Subjects at the Massachusetts Institute of Technology deemed these experiments exempt. After informed consent, participants evaluated the accuracy of these 36 headlines on a scale from 1 (not at all accurate) to 4 (very accurate). In the treatment conditions, participants saw “true” and “false” tags immediately before, during, or immediately after reading. In the control condition, participants rated the headlines alone, with no tags. One week later, all participants judged the same 36 headlines for accuracy, this time with no veracity information. See *SI Appendix* for our full methods and preregistrations.

## Supplementary Material

Supplementary File

## Data Availability

Our preregistrations, materials, and anonymized behavioral data are available on OSF (https://osf.io/nuh4q/). Regression tables and separate analyses of each experiment are also on OSF (https://osf.io/bcq6d/).
