# Supplementary material for: Timing matters when correcting fake news
Source: Proc Natl Acad Sci U S A. 2021 Jan 25;118(5):e2020043118. doi: 10.1073/pnas.2020043118 (PMC7865139; doi:10.1073/pnas.2020043118)
Supplement: Supplementary File [file pnas.2020043118.sapp.pdf]

Supporting Information

for

Timing Matters When Correcting Fake News

Method

Participants. In Experiment 1, 1612 Amazon Mechanical Turk (MTurk) workers (827 women, *M* age = 39.55 years) participated in the first session for compensation. We excluded an additional 14 workers who completed the survey twice. 1350 workers (699 women, *M* age = 40.09 years) returned for the second session. In Experiment 2, 1656 MTurk workers (825 women, *M* age = 38.48 years) participated in the first session for compensation. We excluded an additional 15 workers who completed the survey twice. 1333 workers (675 women, *M* age = 39.18 years) returned for the second session.[1]

Design. We manipulated the timing of corrections (before, during, after, none) between subjects.

Materials. We selected 18 true headlines from mainstream news outlets that contained no factual errors. We chose 18 false headlines that Snopes.com, a third-party website that fact checks news, identified as fabricated (see Appendix). According to a pilot study (N = 2008 Amazon Mechanical Turk and 1988 Lucid workers), half of the headlines favored conservative views, while the other half favored liberal views. True and false headlines seemed equally familiar to pilot participants (true $M = 2.46$, $SD = 0.19$, false $M = 2.71$, $SD = 0.07$, on a 3-pt scale) and contained a similar number of words (true $M = 10.44$, $SD = 2.25$, false $M = 12.39$, $SD = 4.00$). Fake news is often highly implausible, so we did not artificially balance the believability of the true and false headlines.

Procedure. During the *exposure phase*, participants evaluated the accuracy of 36 headlines, on a scale from 1 (*not at all accurate*) to 4 (*very accurate*). In the treatment conditions, participants saw "true" and "false" tags immediately before, during, or immediately after reading. Instructions explained that corrections came from Snopes, the oldest and largest fact-checking site. In the control condition, participants rated the headlines alone, with no tags. Experiment 2 added a 3s break between trials, to ensure that participants understood which headline each fact check corresponded to in the prebunking condition. Then all participants completed the *cognitive reflection test* – eight problems (e.g., *If you're running a race and you pass the person in second place, what place are you in?*) with intuitive, but incorrect, answers (e.g., *first place*; 1). Finally, participants answered five questions that tapped their *political knowledge* (e.g., *Whose responsibility is it to nominate judges to federal courts?*) and indicated their own partisanship (*Democrat, Republican, Independent*). One week later, participants completed the *judgment phase*. They judged the same 36 headlines for accuracy, this time with no veracity information.

Analyses

The alpha level was set at .05 for all statistical tests. Given our large sample, we also report standardized *p* values that control Type I error rates ($p_{stan} = p(\sqrt{N}/100)$, 2, 3). We analyzed accuracy ratings one week after exposure using linear regression with robust standard errors clustered on subject and headline. We included dummies for each treatment condition, a dummy for headline veracity (0 = false, 1 = true), and the interaction between veracity and the treatment dummies as independent variables. The coefficient for a treatment condition reflects the extent to which belief differed from the control condition. We preregistered this analysis approach separately for Experiment 1 and Experiment 2. For increased power, we instead aggregate the data from both experiments and include a study dummy and its interaction with veracity; our key

results persist when analyzing each experiment separately. We also ran preregistered analyses testing whether CRT performance or political knowledge moderated the treatment effects.[2]

Footnotes

[1] Some workers started the study but did not finish (Experiment 1: $n = 86$ for session 1, $n = 8$ for session 2; Experiment 2: $n = 119$ for session 1, $n = 12$ for session 2).

[2] Given the short report format, we do not report the results of the preliminary analyses we preregistered that collapse across treatments. We also preregistered separate analyses for belief in false headlines and for discernment, but both analyses can be conducted using the same model (i.e., the reported coefficients on the treatment dummies give the same information as the preregistered model of belief in false headlines).

References

1. S. Frederick, Cognitive reflection and decision making. *J Econ Perspect* **19**, 25-42 (2005).
2. I. J. Good, Comments, conjectures, and conclusions. *J Stat Comput Simul* **16**, 65-75 (1982).
3. D. Lakens, Justify your alpha by decreasing alpha levels as a function of the sample size. The 20% Statistician. http://daniellakens.blogspot.com/ 2018/12/testing-whether-observed-data-should.html. Accessed 11 November 2020.

Appendix

All headlines are available with accompanying header images on OSF.

False Headlines

CORONER'S REPORT: Woman Found on Clinton Estate was Dead 15 Years, Suffered Torture and Malnutrition

Ahead of His Possible Arrest, Jared Kushner Secretly Leaves the Country

BIG Democrat Just Hailed Out of Disney World in Handcuffs Screaming 'I Can Do Whatever I Want!'

BREAKING: Over 500 'Migrant Caravaners' Arrested with Suicide Vests

Denzel Washington: With Trump We Avoided War with Russia and Orwellian Police State

Hispanic Woman Claims, "Donald Trump Paid Me for Sex in Cancun, This is Our Love Child"

Kenya: Authorities Release Barack Obama's "Real" Birth Certificate

Maine House Democrats Vote to Allow Female Genital Mutilation

Michelle Obama Says She Plans to Run Against Trump in 2020 - With Barack as Her V.P.!

Nancy Pelosi's Son Arrested for Murder

Trump Pays Rudy Giuliani $130,000 to Stay Silent from Now On

Trump Reveals Which Democratic President was Also KKK Member, Liberals in Meltdown Mode

Trump Threatens to Cancel Visit to Israel because "The McDonald's There Doesn't Have a Bacon Cheese Big Mac"

President Trump Readies Deportation of Melania after Huge Fight at White House

Trump Wants to Deport American Indians to India

Trump's Top Republican Rival Surrenders, Endorses Donald for 2020

Trump's Top Scientist Pick: "Scientists are Just Dumb Regular People That Think Dinosaurs Existed and the Earth is Getting Warmer"

W.H. Staffers Defect, Releasing Private Tape Recording That Has Trump Silent

True Headlines

Anger in France, Britain Over Trump's Gun Law Speech

Border Patrol Chief on Migrant Crisis: We're Seeing 'Increase After Increase," No Signs of Slowing

At Trump's Big-City Hotels, Business Dropped as His Political Star Rose, Internal Documents Show

Conservative Groups Form Coalition to 'Aggressively' Oppose Socialized Medicine in US

Georgia Representative: Democrats are 'Blind with Rage' in Their Desire to Impeach President Trump

Centrists Warn of 'Slippery Slope' After Democrats Skirt Rules to Fund Agenda

He's Making Things Happen.' Trump Fans Rally in Washington, D.C.

Investment Boom from Trump's Tax Cut Has Yet to Appear

Ex-White House Ethics Chief: Trump's Mar-a-Lago is a 'Symbol of Corruption'

McDaniel: 'I Love My Uncle' Romney, but GOP Should Unite Behind Trump

Rep. Seth Moulton Slams Joe Biden's Pro-Iraq War Vote

U.S. Stocks Tumble After Trump Announces New Import Tariffs

Trump Admits His Cabinet Had 'Some Clinkers'

Trump to Donate $1 Million to Texas Recovery

Trump Exaggerates Mueller Team's Ties to Obama and Democrats

Trump Supporter Arrested After Allegedly Threatening to Kill Members of Congress

Trump Vows Proposed Tax Cuts Will Benefit Middle Class

Summer Zervos, Trump Accuser, Subpoenas 'The Apprentice' Recordings
